# Association between body weight misperception and snacking patterns among adolescents: a population-based cross-sectional study

**DOI:** 10.1186/s12889-023-17316-w

**Published:** 2023-12-21

**Authors:** Ke Jiang, Yu Zhang, Changxiao Xie, Tiankun Wang, Lingxi Zhao, Wen Zhao, Zumin Shi, Manoj Sharma, Yong Zhao

**Affiliations:** 1 School of Public Health, Chongqing Medical University, Chongqing, 400331 China; 2 Research Center for Medicine and Social Development, Chongqing Medical University, Chongqing, China; 3 Research Center for Public Health Security, Chongqing Medical University, Chongqing, China; 4 Department of Nutrition and Food Hygiene, West China School of Public Health, Sichuan University, Sichuan, China; 5 Human Nutrition Department, College of Health Sciences, QU Health, Qatar University, Dohe, Qatar; 6 Department of Social and Behavioral Health, School of Public Health, University of Nevada, Las Vegas (UNLV), Las Vegas, NV United States; 7 Department of Internal Medicine, Kirk Kerkorian School of Medicine, University of Nevada, Las Vegas (UNLV), Las Vegas, NV United States; 8 Chongqing Key Laboratory of Child Nutrition and Heath, Children ’s Hospital of Chongqing Medical University, Chongqing, China; 9 Chongqing Health Center for Women and Children, Women and Children’s Hospital of Chongqing Medical University, Chongqing, China; 10 Department of Clinical Nutrition, West China Second Hospital, Sichuan University, Chengdu, China; 11 Key Laboratory of Birth Defects and Related Diseases of Women and Children, Ministry of Education, Chengdu, China

**Keywords:** Body weight misperception, Snacking patterns, Adolescents, Healthy eating, Nutrition survey

## Abstract

**Background:**

Unhealthy snacking behaviors and body weight misperception are both significant concerns in adolescent health. Weight misperception are common among youth and may influence their motivation to engage in health-related behaviors, however, the effect on snacking patterns choice remains unclear. Our study aimed to examine the relationship between body weight misperception and snacking pattern choice among school adolescents.

**Methods:**

A cross-sectional study was conducted using an online survey platform. Body weight misperception was defined based on perceived body weight and true weight. Snack intake was measured using a qualitative food-frequency questionnaire. Factor analysis was used to identify snacking patterns, and multiple linear regression was employed to examine the association between body weight misperception and snacking patterns.

**Results:**

190,296 students with the average age of 13.3 ± 1.0 years was included, and 44.5% of students misperceived their weight. Overestimation was more prevalent than underestimation. Two snacking patterns, namely a high-calorie snacking pattern and a healthy snacking pattern, were identified with eigenvalues > 1. Weight underestimation was positively linked to high-calorie snacking pattern scores for both normal weight students (β: 0.16, 95% CI: 0.11, 0.21) and students with overweight/obesity (β: 0.44, 95% CI: 0.35, 0.52), and to healthy snacking scores for students with overweight/obesity (β: 0.28, 95% CI: 0.22, 0.33), but negatively linked to healthy snacking pattern scores for normal weight students (β: -0.12, 95% CI: -0.15, -0.09). Conversely, weight overestimation was negatively linked to both high-calorie and healthy snacking pattern scores for normal weight students (β: -0.07, 95% CI: -0.11, -0.04 and β: -0.13, 95% CI: -0.15, -0.10), but positively linked to healthy snacking scores for underweight students (β: 0.15, 95% CI: 0.08, 0.21). Interactions were found between sex, grade, accommodation, only child, primary guardians, parental education level and weight misperception to snacking patterns.

**Conclusions:**

Adolescents with normal weight and overweight/obesity who misperceived their weight exhibited less healthy snacking patterns, whereas underweight students who misperceived their weight displayed healthier snacking patterns. Comprehensive programs are crucial to educate and guide adolescents in understanding their weight status and making healthier snack choices, involving families, schools, and society.

**Supplementary Information:**

The online version contains supplementary material available at 10.1186/s12889-023-17316-w.

## Introduction

Adolescence, typically spanning from ages 10 to 19, represents a critical transitional period between childhood and adulthood [1]. It is a time of intense physical, psychological, and social development, laying the foundation for lifelong health and well-being [2]. However, alarming trends in childhood obesity have raised significant concerns regarding the health of adolescents. According to estimates by the World Obesity Federation, 206 million children and adolescents with obesity aged 5 − 19 years in 2025, and 254 million in 2030 [3]. Similarly, the Report on Nutrition and Chronic Diseases in China (2020) revealed that nearly 20% of Chinese children and adolescents with overweight or obesity aged 6 to 17, with boys exhibiting higher rates than girls [4]. Adolescent obesity, particularly during puberty, has been linked to a host of physical and mental health issues, including depression [5] and cardiovascular disease [6].

The association between physical health and lifestyle behaviors, particularly dietary habits and exercise, is well established [7]. Unhealthy eating behaviors may lead to energy imbalance and obesity. In China, snacking has become a prevalent dietary habit among children and adolescents [8]. Snacking refers to the consumption of food and drink outside of meal times, and is a growing area of concern in public health. While snacking can provide an additional source of energy, the impact on body weight needs to be considered in terms of frequency, portion size, and type of snacking consumed [7, 9]. Studies have demonstrated that high consumption of ultra-processed foods is strongly linked to adolescent and adult obesity [10, 11], oral health problems [12], and an increased risk of cardiovascular disease [13]. Furthermore, a diet high in ultra-processed foods may increase the risk of non-communicable diseases, while unprocessed or coarse processed foods may reduce the risk [14]. Therefore, interventions aimed at improving snack choices could prove effective in addressing the public health challenge of obesity [15].

Body image is a complex, multidimensional construct that encompasses an individual's self-perception and sensory experiences of their body, including their estimation of body size and attitudes toward their physical appearance [16]. Body weight misperception refers to the discrepancy between an individual's perceived weight and their actual weight [17]. Research has established a clear association between body weight misperception and eating behavior [18]. Perception of weight influences adolescents' lifestyle behaviors [19], including their eating habits and mental health outcomes [20]. For example, adolescents who overestimated their weight status are more likely to take behaviors for managing their weight compared to those who accurately perceive their weight status [21]. Notably, there was also a correlation between weight perception and high-calorie snacking behavior [22], with study indicating that adolescents who misperceive their weight tend to consume more snacks [17].

The majority of research on adolescent weight has concentrated on the association between snacking behavior and weight outcomes [23, 24]. However, the impact of weight perception on snacking intake, especially snacking patterns among Chinese adolescents, remains poorly understood. Thus, the aim of this study was to examine the association between body weight misperception and snacking patterns among school adolescents.

## Methods

### Study Design and Sample Collection

This cross-sectional study was conducted between December 2, 2021, and December 15, 2021, using the online survey platform “Questionnaire Star”. With the support of the Chongqing Municipal Education Commission, we adopted the convenient sampling method and selected 310 junior middle schools in 41 districts and counties of Chongqing as the survey sites. The questionnaire link or QR code was sent to the WeChat work groups of these schools. The teacher in charge forwarded the instructions and questionnaire to the parents through WeChat group of grades 7, 8, and 9. With the informed consent of parents and students, students filled in the questionnaire anonymously and independently on weekends or at home after school.

In total, 190,296 questionnaires from grades 7, 8, and 9 were included for analysis after excluding 11,855 outliers and missing data. This study was approved by the Ethics Committee of Chongqing Medical University. The informed consent form was at the beginning of the questionnaire, and the respondents gave their informed consent before they started to fill in the questionnaire.

### Body weight misperception definition

Body weight and height measurements were self-reported. The four body mass index (BMI) categories of normal, underweight, overweight and obese were derived by using the ‘zbmicat’ STATA function, which uses age- and gender-adjusted BMI cut-offs from the International Obesity Taskforce (IOTF) [25]. Based on the survey data from Brazil, Great Britain, Hong Kong, the Netherlands, Singapore, and the United States, the percentile corresponding to a child's BMI curve passing through the adult cut-offs points at age 18, i.e. BMI of 18.50, 25.00 and 30.00 kg/m^2^, is defined as the cut-offs for underweight, overweight and obesity of different ages and sex from 2 to 18 years old [26]. Weight perception was assessed by the question “How would you rate your weight? (1- underweight, 2- normal weight, 3- overweight/obese)”. Body weight misperception was assessed by comparing participants' actual weight status to their self-perceived weight status. Students' self-perceived weight status was categorized as underestimation (self-perceived weight was lower than true BMI classification), correct (self-perceived weight equaled to true BMI classification) and overestimation (self-perceived weight was higher than true BMI classification).

### Snack intake assessment

Intake of snacks was collected using a qualitative food frequency questionnaire (FFQ) based on China Children and Youth Snacks Guide [27]. The FFQ was revised by epidemiologists, statisticians, nutrition, and child health experts to ensure the validity and reliability of the questionnaire content, and has been validated in Chinese children [28]. The definition of snacks was written at the top of the FFQ There were 22 snack items (10 snack groups) in FFQ (Supplementary Table 1). Students were asked to recall the frequency of these 22 snacks intake over the past week according to a 5-grade scale (seven times a week / five to six times a week / three to four times a week / once or twice a week/ never or less than once a week). In the analysis, the consumption was recoded into times per week.

### Covariates

We considered the following demographic characteristics as covariates: age, sex (boy /girl), ethnicity (Han / Miao / Tujia / Hui /others), grade (grade 7 / grade 8 / grade 9 ), accommodation (school / home), residence (urban/rural), the only child (yes / no), primary guardians (parents: parents as primary guardians only / grandparents: grandparents as primary guardians only / mixed: both parents and grandparents are primary guardians /others: with other guardians involved), parents’ education (low: junior high school and below / medium: senior high school or technical secondary school / high: college or bachelor’s degree and above), and BMI categories (underweight / normal-weight / overweight / obese).

### Statistical analysis

Demographic data were represented as the means ± standard deviations (SD) for metric variables and frequencies and percentages (%) for categorical variables. Differences in means (MD) for continuous variables were analyzed using Student’s t-tests or ANOVA, while differences in categorical variables were analyzed using the χ^2^ test. A factor analysis was performed to identify snacking patterns. The results of Kaiser-Meyer-Olkin Measure (KMO) = 0.96 and Bartlett's test of sphericity *p* < 0.01 confirmed the sample adequacy of the factor analysis [29]. Orthogonal rotation (Varimax rotation) was used to help interpretation of the identified factors. Snacking patterns were extracted with eigenvalues > 1, and snack items were found to be strongly associated with the snacking pattern at absolute factor loadings of ≥ 0.40. Snacking patterns were named based on the interpretation of foods with high absolute factor loadings for each snacking pattern [29]. To assess the degree of alignment between each child's diet and the specific snacking pattern, scores for each snacking pattern for each student were calculated by summing all standardized snack items intake frequency multiplied by their correspondent factor loadings, and then multiplying by the square root of the eigenvalue for each snacking pattern [30]. It should be noted that, due to the presence of snack items with negative factor loadings, the scores could potentially be < 0. Consequently, each child was assigned a score for each snacking pattern, where higher scores indicated a stronger correspondence with the snacking pattern.

The relationship between body weight misperception and snacking patterns (pattern scores) was examined using multiple linear regression models. Model 1 adjusted for age, and model 2 further adjusted for sex, ethnicity, grade, accommodation, residence, an only child, primary guardians, and parents’ education. In the subgroup analyses, the multiplicative interaction between body weight misperception and covariates (sex, ethnicity, grade, accommodation, residence, the only child, primary guardians, and parents’ education) was examined by adding the product of the variables in the regression model.

All statistical analyses and data management were performed in STATA/MP (version 17.0 College Station, TX, USA). A two-tailed *p*-values < 0.05 were considered statistically significant for all analyses.

## Results

### Sample description

A total of 190,296 students from Chongqing were surveyed and included in the analysis. Table 1 presents the demographic characteristics of the sample, which had an average age of 13.3 ± 1.0 years, with a nearly equal proportion of males and females (50.2% vs. 49.8%). The majority of students were in grade 7 (35.6%) with fewer in grade 8 (32.5%) and grade 9 (31.9%), and most were Han Chinese (95.8%). The majority of students lived in urban areas (57.4%) and 44.6% were in school accommodation. Their parents' education level was mostly junior high school (fathers: 64.6%; mothers: 66.0%). The majority were not the only child in their family (76.7%), and the primary guardians were parents (68.0%), grandparents (10.2%), mixed (14.2%), and others (7.5%). The proportion of students categorized as normal, underweight, overweight/obese was 71.3%, 10.5%, 18.2%, respectively.

**Table 1:** Basic demographic characteristics based on actual BMI categories

Factor	Total	Underweight	Normal	Overweight/obsess	*p*-value
N	190296	19888 (10.5%)	135749 (71.3%)	34659 (18.2%)	
Age, mean (SD)	13.3 (1.0)	13.4 (1.0)	13.3 (1.0)	13.2 (1.0)	< 0.001
Sex					< 0.001
Male	95615 (50.2%)	10209 (51.3%)	64069 (47.2%)	21337 (61.6%)	
Female	94681 (49.8%)	9679 (48.7%)	71680 (52.8%)	13322 (38.4%)	
Ethnicity					< 0.001
Han	182340 (95.8%)	19117 (96.1%)	129917 (95.7%)	33306 (96.1%)	
Miao	1885 (1.0%)	161 (0.8%)	1393 (1.0%)	331 (1.0%)	
Tujia	4458 (2.3%)	408 (2.1%)	3310 (2.4%)	740 (2.1%)	
Hui	130 (0.1%)	11 (0.1%)	91 (0.1%)	28 (0.1%)	
Others	1483 (0.8%)	191 (1.0%)	1038 (0.8%)	254 (0.7%)	
Grade					< 0.001
Grade 7	67750 (35.6%)	7251 (36.5%)	46107 (34.0%)	14392 (41.5%)	
Grade 8	61908 (32.5%)	6470 (32.5%)	44603 (32.9%)	10835 (31.3%)	
Grade 9	60638 (31.9%)	6167 (31.0%)	45039 (33.2%)	9432 (27.2%)	
Accommodation					< 0.001
School	84898 (44.6%)	9257 (46.5%)	62191 (45.8%)	13450 (38.8%)	
Home	105398 (55.4%)	10631 (53.5%)	73558 (54.2%)	21209 (61.2%)	
Residence					< 0.001
Urban	109159 (57.4%)	10161 (51.1%)	77078 (56.8%)	21920 (63.2%)	
Rural	81137 (42.6%)	9727 (48.9%)	58671 (43.2%)	12739 (36.8%)	
Only child					< 0.001
Yes	44277 (23.3%)	4104 (20.6%)	30492 (22.5%)	9681 (27.9%)	
No	146019 (76.7%)	15784 (79.4%)	105257 (77.5%)	24978 (72.1%)	
Primary guardians					< 0.001
Parents	129484 (68.0%)	12894 (64.8%)	92347 (68.0%)	24243 (69.9%)	
Grandparents	19366 (10.2%)	2427 (12.2%)	14007 (10.3%)	2932 (8.5%)	
Mixed	27112 (14.2%)	2840 (14.3%)	19143 (14.1%)	5129 (14.8%)	
Others	14334 (7.5%)	1727 (8.7%)	10252 (7.6%)	2355 (6.8%)	
Father’s education					< 0.001
Low	122957 (64.6%)	13629 (68.5%)	88142 (64.9%)	21186 (61.1%)	
Medium	35712 (18.8%)	3487 (17.5%)	25271 (18.6%)	6954 (20.1%)	
High	23097 (12.1%)	1782 (9.0%)	16338 (12.0%)	4977 (14.4%)	
Unknow	8530 (4.5%)	990 (5.0%)	5998 (4.4%)	1542 (4.4%)	
Mother’s education					< 0.001
Low	125572 (66.0%)	13942 (70.1%)	90020 (66.3%)	21610 (62.4%)	
Medium	32384 (17.0%)	2973 (14.9%)	22969 (16.9%)	6442 (18.6%)	
High	21084 (11.1%)	1608 (8.1%)	14808 (10.9%)	4668 (13.5%)	
Unknow	11256 (5.9%)	1365 (6.9%)	7952 (5.9%)	1939 (5.6%)	

Data are presented as mean (SD) for continuous measures, and n (%) for categorical measures

### Body weight misperception by actual BMI categories and sex

**Fig. 1:**
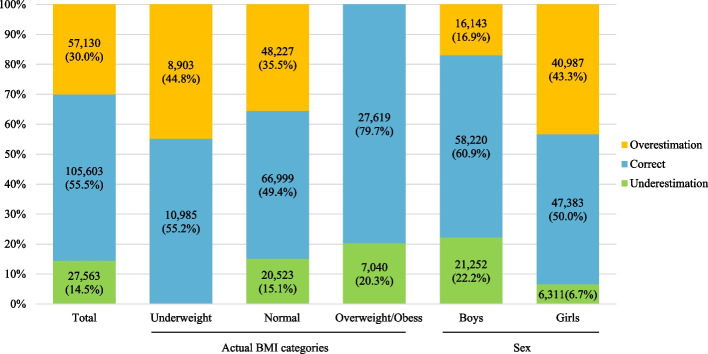
Comparison of body weight misperception by actual BMI categories and sex

The misperception of body weight across different actual BMI categories and sex are illustrated in Fig. 1. The findings reveal a higher prevalence of weight overestimation among students compared to weight underestimation (30.0% vs. 14.5%). Notably, a significant proportion of underweight students (44.8%) tended to overestimate their weight. Among students with a normal weight, more than half (50.6%) demonstrated weight misperception, with 35.5% overestimating and 15.1% underestimating their weight. In contrast, students categorized as overweight or obese displayed a lower likelihood of weight misperception, with only 20.3% underestimating their weight. Furthermore, the rate of weight misperception was found to be lower among boys compared to girls (39.1% vs. 50.0%). Boys exhibited a higher rate of underestimating their weight (22.2%), whereas girls had a higher rate of overestimating their weight (43.3%).

### Snacking patterns identifying

Two snacking patterns were extracted by factor analysis named (1) high-calorie snacking pattern (characterized by high intake of potato chips and French fries; fried meat skewers; odd taste beans and marinated bean curd; high-sugar drinks; puffed food, cream cake, chocolate pie, and spicy gluten; sugar-coated nuts; hawthorn juice, iced tea, and Yakult; condensed milk; marshmallow, milk candy, fruit candy, and chocolate, etc.) and (2) healthy snacking pattern (characterized by high intake of boiled corn, unsweetened oats, and whole wheat bread; soybean milk and roasted soybeans; peanuts, melon seeds, and other nuts; fresh milk and yogurt; freshly squeezed fruit and vegetable juice; fresh fruits and vegetables; poached eggs; roasted sweet potatoes, steamed mashed potatoes, etc.). The two snacking patterns explained 39.6% and 18.8% of the snack intake variances respectively. Snack items at absolute factor loadings of ≥ 0.40 are shown in Table 2.

**Table 2:** Snacking patterns and component loadings extracted by factor analysis

	Component 1	Component 2
Snack items	High-calorie snacking pattern	Healthy snacking pattern
Potato chips and French fries	0.854	
Fried meat skewers and other fried products	0.838	
Odd taste beans and marinated bean curd	0.838	
Coke, Sprite, milk tea and other high-sugar drinks	0.820	
Dark chocolate	0.799	
Puffed food, cream cake, chocolate pie, spicy gluten	0.796	
Fried flour-coated peanut and other sugar-coated nuts	0.784	
Hawthorn juice, iced tea, Yakult and other sugary drinks	0.752	
Condensed milk	0.737	
Marshmallow, milk candy, fruit candy and chocolate	0.726	
Dried groundnuts and sweet potato balls	0.676	0.462
Cheese and milk flakes	0.600	0.484
Roasted sweet potatoes, steamed mashed potatoes and other coarse processed products	0.566	0.522
Dried beef, dried pork, ham sausage and other processed meat products	0.521	
Ordinary cakes and cookies	0.476	0.433
Boiled corn, unsweetened oats, and whole wheat bread		0.717
Soybean milk and roasted soybeans		0.640
Peanuts, melon seeds and other nuts		0.628
Fresh milk and yogurt		0.622
Freshly squeezed fruit and vegetable juice (no extra sugar added)	0.430	0.608
Fresh fruits and vegetables	-0.431	0.598
Poached eggs		0.585
% Variance Explained	39.596	18.810

Snack items considered to be strongly associated with the snacking pattern at absolute factor loadings of ≥ 0.40

### Association between body weight misperception and two snacking pattern scores

The results of the multiple linear regression are shown in Table 3. Following adjustment for potential covariates, weight underestimation was positively associated with high-calorie snacking pattern scores among normal weight students (β: 0.16, 95% CI: 0.11, 0.21) and students with overweight/obesity (β: 0.44, 95% CI: 0.35, 0.52), and was positively associated with healthy snacking pattern scores among students with overweight/obesity (β: 0.28, 95% CI: 0.22, 0.33), but was negatively associated with healthy snacking pattern scores among normal weight students (β: -0.12, 95% CI: -0.15, -0.09). Additionally, weight overestimation was negatively associated with high-calorie snacking pattern scores and healthy snacking pattern scores among normal weight students (β: -0.07, 95% CI: -0.11, -0.04 and β: -0.13, 95% CI: -0.15, -0.10), but was positively associated with healthy snacking pattern scores among underweight students (β: 0.15, 95% CI: 0.08, 0.21).

**Table 3:** Multiple linear regression analysis of associations between body weight misperception and snacking patterns

Snacking patterns		Underestimation vs. Correct		Overestimation vs. Correct	
		Normal weight	Overweight/ Obese	Normal weight	Underweight
High-calorie snacking pattern	Model 1^a^	0.22 (0.18, 0.27)^**^	0.52 (0.43, 0.60)^**^	-0.09 (-0.12, -0.06)^**^	0.03 (-0.06, 0.11)
	Model 2^b^	0.16 (0.11, 0.21)^**^	0.44 (0.35, 0.52)^**^	-0.07 (-0.11, -0.04)^**^	0.06 (-0.02, 0.15)
Healthy snacking pattern	Model 1^a^	-0.04 (-0.07, 0.01)^*^	0.29 (0.23, 0.35)^**^	-0.23 (-0.26, -0.21)^**^	0.08 (0.02, 0.14)^**^
	Model 2^b^	-0.12 (-0.15, -0.09)^**^	0.28 (0.22, 0.33)^**^	-0.13 (-0.15, -0.10)^**^	0.15 (0.08, 0.21)^**^

^a^Model 1 adjusted for age

^b^Model 2 adjusted for age, sex, ethnicity, grade, accommodation, residence, only child, primary guardians and parents’ education

Beta and 95% CI were derived from multiple linear regression

^*^P < 0.05, ^**^P < 0.01

### Subgroup analyses

There were significant interactions between weight misperception and sociodemographic factors (sex, grade, accommodation, only child, primary guardians, parental education level) in relation to snacking patterns. Notably, there was an interaction between sex and weight misperception in relation to both snacking patterns. The association between underestimation of weight and high-calorie snacking pattern was stronger among females than males. The inverse association between overestimation of weight and both snacking patterns was only significant among females but not males (Table 4).

**Table 4:** Subgroup analyses of associations between body weight misperception and sociodemographic factors in relation to snacking patterns

	High-calorie snacking pattern				Healthy snacking pattern			
Factor	Underestimation vs. Correct	*P* ^1^	Overestimation vs. Correct	*P* ^1^	Underestimation vs. Correct	*P* ^1^	Overestimation vs. Correct	*P* ^1^
Sex		**0.008**		**0.000**		**0.003**		**0.000**
Male	0.20 (0.15, 0.25)^**^		0.02(0.00, 0.03)		-0.02(-0.05, 0.02)		0.06(0.02, 0.09)^**^	
Female	0.32 (0.25, 0.40)^**^		-0.04(-0.08, 0.00)^*^		0.06(0.01, 0.11)^*^		-0.08(-0.11, -0.05)^**^	
Ethnicity		**0.012**		0.301		0.148		0.451
Han	0.22(0.18, 0.26)^**^		-0.01(-0.04, 0.02)		-0.01(-0.04, 0.02)		-0.04(-0.06, -0.02)^**^	
Miao	0.24(-0.13, 0.61)		-0.17(-0.46, 0.13)		0.13(-0.14, 0.40)		-0.17(-0.38, 0.05)	
Tujia	0.57(0.31, 0.84)^**^		-0.16 (-0.36, 0.04)		0.15(-0.03, 0.34)		-0.04(-0.18, 0.10)	
Hui	-1.70(-3.34, -0.06)^*^		-0.78(-2.36, 0.80)		-0.30(-1.27, 0.67)		-0.72(-1.65, 0.21)	
Others	0.03(-0.42, 0.48)		0.04(-0.29, 0.37)		0.19(-0.13, 0.51)		0.04(-0.19, 0.28)	
Grade		0.784		0.508		0.047		**0.000**
Grade 7	0.22(0.16, 0.29)^**^		-0.05(-0.11, 0.01)		0.04(0.00, 0.08)		0.04(0.00, 0.08)^*^	
Grade 8	0.24(0.17, 0.31)^**^		0.01(-0.05, 0.06)		-0.05(-0.10, 0.00)^*^		-0.05(-0.09, -0.01)^**^	
Grade 9	0.21(0.14, 0.29)^**^		0.00(-0.05, 0.06)		-0.01(-0.06, 0.05)		-0.09(-0.12, -0.05)^**^	
Accommodation		0.701		0.935		0.651		**0.001**
School	0.22(0.17, 0.28)^**^		0.00(-0.05, 0.04)		-0.01(-0.05, 0.03)		-0.07(-0.10, -0.03)^**^	
Home	0.23(0.17, 0.28)^**^		-0.03(-0.07, 0.01)		0.00(-0.04, 0.04)		-0.02(-0.05, 0.01)	
Residence		**0.002**		0.231		0.666		0.137
Urban	0.18(0.13, 0.23)^**^		-0.02(-0.06, 0.02)		0.00(-0.03, 0.04)		-0.03(-0.06, 0.00)	
Rural	0.28(0.22, 0.34)^**^		-0.01(-0.06, 0.03)		-0.02(-0.06, 0.03)		-0.05(-0.09, -0.02)^**^	
Only child		0.822		0.046		0.346		**0.000**
Yes	0.24(0.16, 0.32)^**^		0.01(-0.06, 0.08)		-0.01(-0.06, 0.04)		0.04(0.00, 0.09)	
No	0.22(0.17, 0.26)^**^		-0.02(-0.06, 0.01)		0.00(-0.03, 0.03)		-0.06(-0.09, -0.04)^**^	
Primary guardians		0.224		0.184		**0.015**		**0.022**
Parents	0.24(0.19, 0.29)^**^		-0.01(-0.05, 0.03)		0.02(-0.01, 0.06)		-0.04(-0.07, -0.02)^**^	
Grandparents	0.22(0.10, 0.34)^**^		-0.07(-0.16, 0.02)		-0.11(-0.20, -0.02)^*^		-0.07(-0.14, -0.01)^*^	
Mixed	0.22(0.11, 0.32)^**^		-0.01(-0.09, 0.07)		-0.07(-0.14, 0.00)		0.01(-0.05, 0.07)	
Others	0.08(-0.06, 0.21)		-0.09(-0.20, 0.02)		0.00(-0.10, 0.10)		-0.03(-0.11, 0.05)	
Father’s education		0.183		0.656		0.055		**0.000**
Low	0.24(0.19, 0.29)^**^		-0.02(-0.05, 0.02)		0.01(-0.02, 0.05)		-0.05(-0.08, -0.02)^**^	
Medium	0.20(0.10, 0.29)^**^		-0.05(-0.12, 0.03)		-0.07(-0.13, -0.01)^*^		0.00(-0.05, 0.05)	
High	0.18(0.06, 0.30)^**^		-0.07(-0.17, 0.03)		0.02(-0.05, 0.10)		0.08(0.02, 0.14)^*^	
Unknow	0.27(0.09, 0.44)^**^		0.07(-0.05–0.19)		-0.02(-0.17, 0.12)		-0.09(-0.19, 0.00)	
Mother’s education		**0.024**		0.414		0.960		**0.000**
Low	0.25(0.20, 0.30)^**^		-0.02(-0.06, 0.02)		-0.01(-0.04, 0.03)		-0.06(-0.08, -0.03)^**^	
Medium	0.17(0.07, 0.27)^**^		-0.04(-0.12, 0.04)		0.00(-0.06, 0.07)		0.05(0.00, 0.11)^*^	
High	0.14(0.02, 0.26)^*^		-0.02(-0.13, 0.08)		0.01(-0.06, 0.09)		0.07(0.00, 0.13)^*^	
Unknow	0.29(0.14, 0.44)^**^		-0.01(-0.12, 0.09)		-0.03(-0.15, 0.09)		-0.12(-0.21, -0.03)^**^	

Model adjusted for age sex, ethnicity, grade, accommodation, residence, only child, primary guardians, and parents’ education

Beta and 95% CI were derived from multiple linear regression

^1^P for interaction. Statistically significant *p*-values are shown in bold

^*^P < 0.05, ^**^P < 0.01

## Discussion

In this study, we explored snacking patterns and their association with body weight misperception in a sample of junior middle school students in Chongqing, China. Two snacking patterns were identified, named high-calorie snacking pattern and healthy snacking pattern. Our results revealed that almost half of the students in our sample misperceived their weight status, with overestimation of weight status being more prevalent than underestimation. This may be due to the aesthetic concept of thinness as beauty, leading many young people to focus on their body shape under social pressure to conform to the idea of thinness [31]. The weight misperception rate in our study was slightly higher than those reported among young adults in Pakistan (42.4%) [32], Mexico (36.9%) [33], Ghana (20.6%) [34], and among adolescents in Korea (34.1%) [35] and Spain (23.5%) [36], but similar to the study among adults in Guangdong, China (50.2%) [37]. These variations may reflect differences in social norms of ideal weight across ethnic and cultural contexts.

However, we all found that boys were more prone to underestimating their weight, while girls were more prone to overestimating their weight. This may be partly attributable to the influence of media on appearance, which tends to promote slimness in girls and influence their weight standards [38], while male students may be less conscious of weight management and may be influenced by traditional beliefs that associate boys' strength with being overweight, thereby ignoring their unhealthy weight [39]. To prevent these risks, firstly, general education about weight perception is essential. Secondly, society needs to work towards changing the norms of skinny women and muscular men into achievable and healthier body shape. Then, we should educate girls and boys to pursue fitness or thinness under the premise of health, and not use sex to solidify the aesthetic. Finally, sex differences should also be taken into account when developing appropriate intervention programs. For example, appropriate youth sports education programs should focus on healthy growth and development rather than physical appearance [40].

Interestingly, our research also found that students categorized as overweight or obese represented a lower likelihood of weight misperception, with only 20.3% underestimating their weight. We surmise the tendency to overestimate weight can explain this phenomenon. Due to the social condemnation and media influence on people with overweight/obesity, few of them think that they is thin or normal, and weight misperception in people with overweight/obesity have only one option: underestimation. In addition, body weight misperceptions, including both underestimation and overestimation, could result in incorrect behaviors about weight control and eating [31]. Weight misperception, in particular overestimation, was related to psychological outcomes such as stress [40]. Therefore, programs and comprehensive interventions on correcting adolescent weight misperceptions to establish correct body image perceptions are necessary for the physical and mental health of teenagers. A contrary to commonly held thoughts study show weight misperception among youth who were overweight or obese predicted lower future weight gain. Thus, we should rigorously examine the efficacy of efforts to correct weight misperception to assess for both intended and unintended consequences [41].

It has been shown that adolescents' actual BMI status affects their self-reported weight and the associated weighting behavior, so different actual BMI classifications were considered in our analysis [42]. Incorrect perceptions of body weight have been linked to both healthy and unhealthy dieting behaviors [35, 43]. And our study revealed that compared to students who correctly perceived their weight, students with normal weight who underestimated their weight were more inclined to the high-calorie snacking pattern. Our findings are consistent with prior research that has reported an association between overestimating body weight and unhealthy snacking behaviors in adolescents [40, 44, 45], which can increase the risk of overweight and obesity. Adolescents with overweight or obesity who underestimated their weight prefer both of the snacking patterns above, indicating that those who misperceived their weight consumed more snacks, regardless of the snack type [46]. A study conducted in Indonesia similarly found that adolescent girls who underestimated their weight were 2.7 times more likely to consume deep-fried crackers compared to those who correctly assessed their weight [19]. But a study of 7–12 years old children in Guangzhou, China, reported that among overweight children, those who accurately perceived their weight had a higher intake of fruits and vegetables than those who underestimated their weight [47]. This difference may be attributed to the fact that the outcome variable in our study was the snacking pattern, which took into account the consumption of other types of crude processed foods, whereas the outcome variable in the Guangzhou study was only the intake of fruits and vegetables.

Meanwhile, many studies have identified the misperception of being with overweight or obesity among adolescents at normal weight as a risk factor for eating behavior disturbances [48, 49]. A study of Iranian children also showed that in comparison with the accurate-weight group, the overestimated-weight groups were less likely to have a daily consumption of sugar-sweetened beverages, sweets, and salty snacks [50]. Although snacking less may help to reduce the risk of obesity, the sequence of weight misperception, body dissatisfaction, and dieting (especially of healthy foods like vegetables, fruits, and milk) may lead to negative eating attitudes, which could increase the risk of various pathologies, including anxiety/depression, psychological distress symptoms, anorexia, and malnutrition [18, 49, 51]. Regarding underweight students, a previous study in Guangzhou, China, found that underweight children with accurate perception had a lower intake of fruits and vegetables than those who overestimated their weight [47]. However, another study in Wuhan, China, showed that adolescents who overestimated their weight were more likely to consume fruits, but less milk and dairy products [45]. Our results highlight the importance of healthy snacks for weight management. Previous studies have concluded whole foods high in protein, fiber, and whole grains (e.g., nuts, yogurt, prunes, and popcorn) enhance satiety when consumed as snacks potentially reducing obesity risk by curbing overconsumption at subsequent meals [52]. Moreover, studies have observed an inverse association between the intake of healthy foods (such as vegetables, fruits, nuts, and pulses or lentils) and BMI [53–55]. The healthy snacking pattern for low-weight children should be promoted among children with normal-weight, overweight and obesity to prevent excessive calorie intake and the risk of various obesity-related non-communicable diseases in adulthood and premature death [56].

In the subgroup analyses, the interaction between several sociodemographic characteristics and body weight misperception to snacking patterns has been found. Specifically, boys who underestimated their weight were found to prefer high-calorie snacking patterns, whereas those who overestimated their weight tended to choose healthy snacks. In contrast, girls who overestimated their weight did not show a specific preference for snack patterns, while those who underestimated their weight tended to reduce their snack intake. Previous studies have shown that boys and girls with the same self-perception of their weight make different foods choices [57–59], but these studies did not focus on snacks. Our results suggest that both boys and girls who underestimate their body weight are at increased risk of obesity due to excessive intake of high-calorie snacks. This is because they believe they are not overweight and continue to consume large quantities of unhealthy snacks without considering the consequences. This can lead to weight gain and an increased risk of obesity, which can have serious health implications for these children [60]. Besides, unlike girls who tended to completely avoid snacks due to their overestimation, boys made more nutritious snack choices, suggesting that boys may have a better understanding of healthy eating habits and are more conscious of the impact of their snack choices on their weight and overall health. However, girls who overestimated their weight may have a distorted body image and could benefit from education and support to help them adopt a healthier relationship with food [61]. By encouraging boys to continue making reasonable snack choices and empowering girls to overcome their negative relationship with food, we can help children maintain a healthy weight and prevent the development of obesity and related health problems.

Then, our results showed that among students who overestimated their weight, students in lower grades were more likely to choose a healthy snacking pattern. This finding is consistent with previous research reporting lower fruit and vegetable consumption and higher intake of energy-dense snacks among younger students [62]. However, students who live in school and non-only child were less likely to choose healthy snacking patterns. This may be attributed to the difficulty in accessing these snacks, such as fresh fruits and vegetables, in the school environment, leading to a lower intake of healthy snacks. Other studies have also highlighted the influence of environmental accessibility, both at home and school, on food choices [62, 63]. Furthermore, non-only child's snack choices may be influenced by their siblings, similar to peer influence [64]. Additionally, among students whose parents were the primary guardians, those who underestimated their weight were more likely to choose healthy snacking patterns, while those who overestimated their weight were less likely to choose healthy snacking patterns. Studies have shown that parents engage in a variety of behaviors (including restrictive and positive guidance) to promote or discourage food consumption behaviors in their children [64, 65]. It is possible that parents of underweight students are concerned about their child's nutritional deficiencies and physical health, leading them to focus more on healthy eating. Conversely, parents of students who overestimate their weight may indulge their children more, resulting in a lower intake of healthy snacks.

Finally, parental education (especially the mother’s education) was also an important factor in the relationship between body weight misperception and snacking patterns. Students with low levels of parental education who underestimated their weight were more likely to choose high-calorie snacks, while students with high levels of parental education who overestimated their weight were more likely to choose healthy snacks. These findings align with previous research indicating that parental education level has a significant impact on children's snack choices, with more educated parents promoting healthier choices for their children [66–68]. Parental education level can influence the availability of healthier snacks in the household and the resources to purchase them [67]. Additionally, parents with higher education levels are more likely to educate their children on the importance of a balanced diet, healthy eating habits, and the consequences of consuming unhealthy snacks [69]. In conclusion, this finding underlines the critical role of parental education level in shaping the eating habits of children and promoting healthy snack choices.

The strengths of our study are obvious. The large sample of over 190,000 students covering all 41 districts and counties of Chongqing makes the study results more representative and generalizable. Besides, we reduced the dimension of the complicated snack intake items and frequency data in FFQ, which simplified the difficulty of data analysis and presentation while retaining the essence of the original information, and made the results easy to understand. However, some limitations of this study are worth noting. First, due to the study’s cross-sectional design, no causal conclusions could be drawn, instead, only associations between weight misperception and snacking pattern were identified. Second, the survey was conducted mainly online, and students' height and weight were based on self-reported values, which may be biased or inaccurate. Third, there is currently a dearth of authoritative snack frequency questionnaires available in China, the FFQ utilized in our study may not have possessed sufficient detail and authority in categorizing snack items. It should be acknowledged that one snack item may belong to both the healthy snack pattern and the unhealthy snack pattern. And the FFQ only collects the frequency of individual snack intake, not the specific amount consumed, which may reduce the ability to assess the quality of snack intake through the FFQ. ​However, some scholars have shown that standardized portion size specifications may not introduce large errors in food intake estimation, and we chose FFQ because our main objective was to obtain "healthy" and "unhealthy" snacking patterns, rather than exact amounts of snacks. There is a need for future studies to design snack frequency questionnaires with more accurate classification of snack items and higher validity. ​Despite the limitations of the study, they do not affect the importance of this study.

## Conclusion

In conclusion, our study found that nearly half of the students misperceived their weight status and overestimation of weight status was more prevalent than underestimation. Adolescents with normal weight and overweight/obesity who misperceived their weight exhibited less healthy snacking patterns, whereas underweight students who misperceived their weight displayed healthier snacking patterns. Furthermore, there were significant interaction between sex, grade, accommodation, only child, primary guardians, parental education level and body weight misperception. To address this, it is important for families, schools, and society to implement comprehensive programs to help adolescents understand their weight status and guide them to make healthier snack choices. Those who underestimate their weight should be encouraged to reduce high-calorie snacks and focus on weight management, while those who overestimate their weight should eat more nutritious snacks instead of avoiding snacks altogether. This will reduce the risk of obesity, chronic diseases in the former, and anorexia and wasting in the latter.

### Supplementary Information

**Additional file 1: Supplementary Table 1. **Snacking Frequency Questionnaire.

## Data Availability

The datasets generated and/or analyzed during the current study are not publicly available due to funding requirements but are available from the corresponding author on reasonable request.
