# Supplementary material for: Association between body weight misperception and snacking patterns among adolescents: a population-based cross-sectional study
Source: BMC Public Health. 2023 Dec 21;23:2550. doi: 10.1186/s12889-023-17316-w (PMC10734079; doi:10.1186/s12889-023-17316-w)
Supplement: Supplementary file 1 — Additional file 1: Supplementary Table 1. Snacking Frequency Questionnaire. [file 12889_2023_17316_MOESM1_ESM.docx]

**Supplementary Table 1.** Snacking Frequency Questionnaire

| Snack groups | Snack items | Seven times a week | Five to six times a week | Three to four times a week | Once or twice a week | Never or less than once a week |
| --- | --- | --- | --- | --- | --- | --- |
| Candy | Marshmallow, milk candy, fruit candy and chocolate |  |  |  |  |  |
|  | Dark chocolate |  |  |  |  |  |
| Meat and eggs | Poached eggs |  |  |  |  |  |
|  | Dried beef, dried pork, ham sausage and other processed meat products |  |  |  |  |  |
|  | Fried meat skewers and other fried products |  |  |  |  |  |
| Grains | Boiled corn, unsweetened oats, and whole wheat bread |  |  |  |  |  |
|  | Ordinary cakes and cookies |  |  |  |  |  |
|  | Puffed food, cream cake, chocolate pie, spicy gluten |  |  |  |  |  |
| Potatoes | Roasted sweet potatoes, steamed mashed potatoes and other coarse processed products |  |  |  |  |  |
|  | Dried groundnuts and sweet potato balls |  |  |  |  |  |
|  | Potato chips and French fries |  |  |  |  |  |
| Beans and bean products | Soybean milk and roasted soybeans |  |  |  |  |  |
|  | Odd taste beans and marinated bean curd |  |  |  |  |  |
| Fresh fruits and vegetables | Fresh fruits and vegetables |  |  |  |  |  |
| Nuts | Peanuts, melon seeds and other nuts |  |  |  |  |  |
|  | Fried flour-coated peanut and other sugar-coated nuts |  |  |  |  |  |
| Dairy and dairy products | Fresh milk and yogurt |  |  |  |  |  |
|  | Cheese and milk flakes |  |  |  |  |  |
|  | Condensed milk |  |  |  |  |  |
| Beverages | Freshly squeezed fruit and vegetable juice (no extra sugar added) |  |  |  |  |  |
|  | Hawthorn juice, iced tea, Yakult and other sugary drinks |  |  |  |  |  |
|  | Coke, Sprite, milk tea and other high-sugar drinks |  |  |  |  |  |

Snacks: Refers to all food and beverages (excluding water) consumed outside the three main meal times of the day.
